# Impressions of a German Hospital: The Eppendorf Hospital in Hamburg

**Published:** 1914-12-19

**Authors:** 


### IMPRESSIONS OF A GERMAN HOSPITAL.

### The Eppendorf Hospital in Hamburg.

BY A RESIDENT MEDICAL OFFICER.

To see something of as many other hospitals as he can, whether at home or abroad, is always a profitable undertaking for a resident medical officer. The trouble entailed is trifling compared with the pleasure of combining with travel practical knowledge of the actual working of such improvements and novelties as he is sure to come across. By devoting a few hours of his leisure to this form of "'busman's holiday" he will be materially strengthening his position as one who speaks with authority when the time comes that he may wish to put forward demands for new material or structural alterations in his own institution at home.

German hospitals are run, as my readers are no doubt aware, on very different administrative lines from those in this country. But to note this difference was not the only objective of my visit to the well-known Eppendorfer Krankenhaus just outside Hamburg. Both Hamburg and Lübeck were Hansa cities of great mercantile renown in the past. Hamburg, of course, has risen again, while Lübeck preserves glorious traces of its former wealth and power, and is a delightful place for tourists.

### The Secretary's Office and Visitors.

German officialism does not offer any hindrance to medical men seeking admission to view the large municipal hospitals, and if one can get in touch with one of the visiting professors all is plain sailing. In any case application at the Bureau is the first step in every hospital. This Bureau is the headquarters of all the administrative departments of the hospital, and controls a greater number of details than the usual secretary's office in this country. The payments by the patients, for instance, require a deal of book-keeping. It will be remembered that all the patients in German hospitals pay, or are paid for; in the latter case either by the equivalent of our Friendly Societies or by the municipality. From the Bureau the visitor will be conducted by one of the porters on his tour of the grounds, or be delivered over to the professor he desires to meet. In many of the largest hospitals, such as the Rudolf Virchow Hospital in Berlin, regular hours for sight-seeing are arranged on certain days, and "conducted tours" form a recognised branch of the hospital's activities. Owing to the very extensive nature of the grounds in which the hospital buildings are usually set, it is quite necessary that parties should be formed of would-be sight-seers to prevent waste of time on the part of the staff.

### The Gardens and Pavilions.

The Eppendorf Hospital is situated well outside the city of Hamburg, in a very pleasantly laid-out suburb. Owing to this remoteness from the centre of the town a large number of ambulance vans are kept constantly employed in conveying patients to and fro. The advantages, however, of the spacious surroundings of the hospital outweigh any disadvantages due to distance.

The hospital proper is laid out, like so many other German *Krankenhäuser*, in numerous pavilions amid lawns, gravel paths, and patches of shady trees. All branches of medical and surgical work are represented, the grounds being divided up into "districts," or groups of pavilions, for surgical, medical, and gynæcological cases, for tuberculosis, for special diseases (eyes, etc.), for confinement cases, and a division for the infectious diseases. This latter district is well separated from the rest, and is surrounded by a wire fence.

In other parts of the grounds are the fine kitchens, the laundries, stables, and engineering departments. The kitchens are splendidly equipped with labour-saving machinery and the latest and most efficient types of cookers and cauldrons. The food is kept hot, during distribution, in jacketed tins, and is conveyed to the various pavilions in vans. The details are similar to those of the method in use at Queen Mary's (the children's) Hospital at Carshalton. Each ward has a small kitchen, in which minor cooking is done and the major meals dealt with for distribution.

### The Wards.

The wards themselves are well constructed, light, and lofty; being isolated, cross-ventilation is everywhere available. The pavilions are nearly all entered at one end, where there is a roomy lobby containing store rooms, sister's room, and a well-equipped clinical laboratory. In the wards the beds are rather more closely placed than is customary in our own hospitals, but the difference in the amount of air space allowed per bed is not great. The bath-room accommodation and w.c. provision are ample and excellent.

The resident medical officers are lodged in a detached house in a very pretty part of the grounds, and their rooms are comfortably, if not luxuriously, furnished. Naturally a good system of telephones is provided, and the hospital exchange is quite a big affair. The *x*-ray department is a very active one, and the instruments and their numerous mechanical accessories are of the latest pattern. A great deal of work in this department is done for the tuberculosis division of the hospital, and the *x*-rays are relied upon to a large extent to supplement the clinical records of the progress of phthisical patients.

A view was obtained of the magnificent hall used for scientific meetings and clinical discussions. This room is equipped with an epidiascope and cinematograph; the lecture tribune, demonstration table, and screens are most practical and convenient, while the desks and seats for the audience are almost too comfortable for such a serious conventicle.

The "Liege-halle" or Wide Verandah.

Returning now to the division for tuberculosis, we find several large pavilions of one storey each, containing from twenty-five to thirty beds. Along one side, facing the south, is a wide covered *Liege-halle,* or very wide verandah, facing a rough lawn bordered by shady trees. On the verandah are placed not only couches and deck-chairs, but several beds, while in suitable weather numbers of patients recline on couches under the trees. In fact, this portion of the hospital was in no way inferior to a sanatorium, except in the matter of walking exercise or graduated labour, which, of course, could not be arranged. The patients under treatment here are, however, not those who could with benefit be allowed exercise; they are all "hospital" cases, and were being treated with a view to getting them fit enough to be transferred to sanatoria. The history boards and temperature charts are carefully kept up to date, and contain elaborate details of condition and treatment. A large proportion of the patients were being treated in accordance with the programme elaborated in connection with the Deycke-Much partial-antigen method of tuberculin administration.

In the pavilions allotted to the infectious fevers the several diseases are strictly isolated, and there are in use many single-bed rooms for observation purposes, besides a certain number of private rooms for better-class patients. The methods of dealing with scarlet fever and diphtheria are in no way superior to those in vogue at home. The scarlet-fever patients seem to be detained rather longer than is now considered necessary in this country. In the diphtheria wards at Eppendorf much smaller doses of antitoxin were given than we are accustomed to. The opinion of the professor in charge is that the large doses not infrequently given in our hospitals are not only useless and wasteful, but entail unnecessary and detrimental efforts on the part of the patient for their elimination.

